# A Multicenter, Double-Blind, Randomized, Placebo-Controlled Phase 2b Trial of Cytisinicline in Adult Smokers (The ORCA-1 Trial)

**DOI:** 10.1093/ntr/ntab073

**Published:** 2021-04-12

**Authors:** Mitchell Nides, Nancy A Rigotti, Neal Benowitz, Anthony Clarke, Cindy Jacobs

**Affiliations:** 1 Los Angeles Clinical Trials, Burbank, CA, USA; 2 Tobacco Research and Treatment Center, Massachusetts General Hospital and Harvard Medical School, Boston, MA, USA; 3 University of California San Francisco, San Francisco, CA, USA; 4 Achieve Life Sciences Inc., Seattle, WA, USA

## Abstract

**Introduction:**

Cytisinicline (known as cytisine), a nicotinic acetylcholine receptor partial agonist, is a smoking cessation aid currently marketed in Central and Eastern Europe using a 1.5-mg/tablet 25-day downward titration schedule. No prior studies have evaluated other doses or administration schedules. This study evaluated the effects of a higher dosage and simplified dosing schedule on drug efficacy and tolerability.

**Methods:**

ORCA-1 was a double-blind, randomized, placebo-controlled clinical trial that provided cytisinicline or placebo tablets plus behavioral support for 25 days. Adult smokers (*>*10 cigarettes daily) committed to quitting smoking were randomized to compare 2 cytisinicline doses (1.5 mg and 3 mg) versus placebo, and 2 administration schedules [downward titration versus 3 times daily (TID)]. Primary outcome was a reduction in expected cigarettes smoked at end of treatment; secondary outcomes were biochemically confirmed 7-day abstinence at Week 4 and continuous abstinence from Weeks 5 to 8.

**Results:**

Among 254 participants, those in cytisinicline arms (regardless of dose or schedule) had greater reductions in cigarettes smoked versus placebo, with differences observed in 3 cytisinicline arms statistically significant versus placebo. All cytisinicline arms had statistically significantly higher abstinence rates at Week 4 versus placebo. Both cytisinicline arms using TID schedules had statistically significantly higher continuous abstinence rates from Weeks 5 to 8 compared with placebo. Participants in the cytisinicline 3-mg TID arm had the highest abstinence rate. There were no safety concerns with either 1.5-mg or 3-mg cytisinicline.

**Conclusion:**

Based on simpler dose scheduling, excellent tolerability, and best-continued abstinence rate, cytisinicline 3-mg TID was selected for future Phase 3 studies.

**Implications:**

Although the 1.5-mg 25-day titration schedule has been marketed in Central and Eastern Europe for decades, this study explored using a higher dosage and a simplified dosing schedule for impact on cytisinicline efficacy and tolerability. Based on these results, a Phase 3 program was initiated using cytisinicline 3-mg tablets on a TID schedule for potential market approval in the United States.

## Introduction

Tobacco smoking contributes to 8 million premature deaths each year worldwide.^1^ Stopping smoking can reduce these health risks and increase life expectancy, but, because tobacco smoking is highly addictive, more than 95% of unaided attempts at cessation fail by 6 months.^2^ Addiction results from nicotine’s interaction with nicotinic acetylcholine receptors (nAChRs) in the central nervous system. A large body of evidence indicates that multiple quit attempts are often needed to achieve long-term tobacco abstinence.^3,4^

Cytisinicline is a plant-based single enantiomer alkaloid [(-)-cytisine] that can be isolated from various plant sources. Its molecular structure has similarities to nicotine, and cytisinicline and nicotine compete for binding to nAChRs.^5–7^ Nicotine is thought to stimulate nAChRs in the ventral tegmental area of the brain, resulting in the release of dopamine in mesolimbic loci, such as the amygdala. Cytisinicline’s mechanism of action as a partial nAChR agonist in treating nicotine addiction is 2-fold: 1) the partial agonism maintains some release of dopamine (although lower than the level stimulated by nicotine) and therefore reduces craving and other withdrawal symptoms, and 2) the partial antagonism reduces nicotine binding, therefore reducing pleasure and other rewarding effects of smoking. Cytisinicline has been approved and used as a smoking cessation medication in Europe since the 1980s,^8,9^ with an administration schedule consisting of 1.5-mg tablets in a downward titration, from 6 tablets/day to 1 tablet/day over a 25-day period. More recent Good Clinical Practice (GCP) studies in Europe and New Zealand have shown cytisinicline is an effective smoking cessation therapy with an excellent safety profile^10,11^ when compared with placebo or nicotine replacement therapy (NRT).

In the United States (U.S.), current Food and Drug Administration (FDA)-approved smoking cessation medications include 5 forms of NRT and 2 non-nicotine medications, bupropion and varenicline. Varenicline was synthetically developed, in part, using (-)-cytisine as a structural starting point.^5^ Thus, varenicline is a partial agonist at nAChRs, similar to cytisinicline. Varenicline has demonstrated greater efficacy for smoking cessation than the nicotine patch or bupropion, when compared in a large double-blind, placebo-controlled randomized trial.^12^ Across cohorts in the trial, the most frequent adverse events (AEs) by treatment group were nausea (varenicline, 25%), insomnia (bupropion, 12%), abnormal dreams (nicotine patch, 12%), and headache (placebo, 10%). Other than nausea, some of the common AEs observed with varenicline treatment include vomiting, abnormal (ie, vivid, unusual, or strange) dreams, insomnia, and headache, which can lead to treatment discontinuation.

Despite its long history of use, cytisinicline has not been approved for marketing outside Europe. For the global population of tobacco smokers for whom smoking cessation is a goal, cytisinicline has the potential to be as effective as varenicline, but with fewer off-target effects in comparison with varenicline. Notably, the incidence of nausea and vomiting has been reported to be lower with cytisinicline treatment^10,11^ in comparison with varenicline.^12^

The ORCA-1 trial (ClinicalTrials.gov: NCT03709823) was designed based on discussions with FDA regarding clinical trial development options for potential market approval in the U.S., including a simpler dosing regimen and a higher cytisinicline dose. A 3 times daily (TID) administration schedule was chosen as a simplified schedule based on the pharmacokinetic profile of cytisinicline and a half-life of 4.8–4.9 hours after a single dose,^13,14^ which should result in participants having adequate plasma concentrations of cytisinicline throughout the day. A 3-mg cytisinicline dose was chosen to be evaluated in addition to the 1.5-mg dose in both schedules. The 3-mg TID daily dose (9 mg) would be comparable to the highest initial daily dose (1.5 mg given 6 times daily) administered in the European marketed titration schedule. The primary endpoint was the reduction in expected cigarette smoking during study treatment, chosen to assess possible effects of the 2 doses and schedules on smoking behavior. A secondary endpoint of continuous abstinence, defined as no cigarettes smoked with biochemical verification for 4 consecutive weeks after treatment, was evaluated to assess for improved quit rates, which is a more important criterion for regulatory approval.

## Methods

The study was reviewed and approved by an independent institutional review board and was conducted in accordance with the Declaration of Helsinki and GCP guidelines as denoted in the International Conference on Harmonisation (ICH) E6 requirements.

ORCA-1 was a double-blind, randomized, placebo-controlled study conducted at 8 sites in the U.S. The study planned to enroll 250 adult daily smokers who failed at least 1 previous attempt to stop smoking with or without therapeutic support and were willing to set a quit date 5–7 days after randomization into the study. Participants were identified via each site’s database of known smokers and/or via local advertisement. After initial telephone screening, participants provided written informed consent and proceeded to complete at least 1 on-site screening visit to assess eligibility. Key eligibility criteria: average of *>*10 cigarettes per day assessed by a 7-day diary during screening, exhaled carbon monoxide (CO) *>*10 ppm, no clinically significant medical comorbidities or abnormal laboratory findings, and no treatment with other smoking cessation medications in the 4 weeks before randomization.

Upon confirmation of all inclusion/exclusion criteria, participants were stratified by body mass index (BMI) and randomized to study treatment arms, with the first day of study treatment initiated the following day (Day 1). A centralized interactive response technology system was used to assign participants to treatment arms according to a pre-generated randomization scheme (block size 10).

This was a double-blind study with regard to cytisine versus placebo dosing. However, participants and site staff were unblinded to the treatment schedule (downward titration versus simplified TID) due to packaging and dose-timing requirements. An independent vendor blinded the individual study drug kits. The trial Sponsor and site personnel did not have access to the treatment assignment for individual participants (except in cases of emergency) until the database was locked and final study analysis was performed. Each participant received 25 days of treatment. Six study treatment arms were evaluated.

Participants were randomized in a 2:2:1 ratio to receive cytisinicline 1.5 mg, cytisinicline 3 mg, or placebo (Supplemental Figure 1). After randomization, each participant was required to attend 11 clinic visits [Day 2, 3, 6, 12, 16, 20 during treatment, at End of Treatment (EOT: Day 27 ± 2), and then weekly post-treatment at Week 5, 6, 7, 8]. All participants received approximately 10 minutes of smoking cessation counseling at each clinic visit provided by experienced counselors. Study drug compliance was assessed by participant completion of a daily dosing diary and by drug accountability, conducted by clinical site personnel at each clinic visit during study treatment.

### Safety Measures

Reporting of AEs began at randomization and continued through the Week 8 visit. Venous blood was obtained at screening, at each visit during treatment, at EOT, and at the Week 8 visit for hematology and serum chemistry assessments by a central laboratory. Electrocardiograms (ECGs) were performed at screening, Day 12 during treatment, and at EOT. Overall safety monitoring was performed by 2 independent Data Safety Monitors.

### Efficacy Measures

During screening, each participant recorded the number of cigarettes smoked daily for 7 consecutive days in a diary to generate a daily average of cigarettes smoked (an inclusion criterion). Once enrolled, the number of cigarettes smoked daily during the study treatment was self-reported in a participant diary. The study’s primary efficacy endpoint was the percentage of expected cigarettes smoked, which was computed for each participant at EOT (cigarette score). The cigarette score was calculated as follows:


$$\begin{array}{l} Cigarette\;Score\;=\frac{100\times N}{R\times T} \end{array}$$

where *N* was the total number of cigarettes smoked over the 25-day treatment period; divided by *R* × *T* where *R* represented the average number of cigarettes smoked daily over the 7-day screening period and T was the number of study treatment days actually recorded in the participant’s diary. Thus, *R* × *T* represented the total number of cigarettes that would have been smoked without intervention over the study treatment days.

Initial quit rate at EOT (Week 4) was defined for a participant as having reported smoking no cigarettes from Day 21 to the Day 27 EOT visit, with an expired air CO reading <10 ppm at the EOT (also defined as Week 4) visit. The timing of this endpoint was defined at the EOT/Week 4 visit as abstinence from Day 21 to 27 to allow participants a grace period of approximately 2 weeks to stop smoking completely after the quit date (Day 5–7). After the study treatment ended, each participant reported whether they had smoked any cigarettes (since the last clinic visit or over the past 7 days) at each of the Week 5, 6, 7, and 8 clinic visits with biochemical verification (expired CO reading <10 ppm). Each site measured expired CO using the Bedfont Micro+Smokerlyzer®, or equivalent, which was performed at screening, randomization, EOT (Week 4), and weekly post-treatment at Week 5, 6, 7, and 8.

Seven-day point prevalence abstinence was evaluated as a binary endpoint at Week 4, 5, 6, and 8. At each visit, abstinence was defined for a participant as having reported not smoking any cigarettes over the previous 7 days with biochemical verification by an expired air CO reading <10 ppm.

Continuous abstinence, defined as no cigarettes smoked from Week 5 to Week 8, was measured for a participant as having reported smoking abstinence (no cigarettes since the last clinic visit and over the previous 7 days) at each clinic assessment from Week 5, 6, 7, to Week 8 with biochemical verification at each weekly assessment. Participants could fail to achieve the abstinence measures in 3 ways: (1) participants could report smoking at any time from Week 5 to Week 8 (either by the participant’s self-report of smoking or CO ≥10 ppm) or (2) participants could have missing follow-up data (e.g., participants lost to follow-up, missing the Week 5 and/or Week 8 clinic visit, or missing both the Week 6 and Week 7 visits) or (3) participants could have missing CO levels required for confirmation at the defined clinic visits.

### Statistical Analysis

As the overall goal of the study was to obtain estimates of effect size for efficacy endpoints and to inform the design of future studies; no formal statistical hypothesis testing was conducted. However, statistical testing was performed with p-values and 95% confidence intervals (CIs). The p-values were interpreted as an assessment of the role of chance (small p values indicating a small likelihood that the observed effect was due to chance) and used qualitatively for next-step decisions.

Overall reduction in daily cigarette smoking was chosen as the primary efficacy outcome to assess for possible differences in dosing and schedules, when such factors might have different daily effects on smoking behavior during the 25-day treatment period. The sample size was computed based on daily cigarettes smoked from a previous Phase 1/2 study and for achieving narrow CIs for the primary comparison estimates from the Phase 1/2 study. The standard deviation from the daily cigarettes smoked in the Phase 1/2 study was found to be approximately 17. Based on this standard deviation, the estimated between-arm differences in the primary comparisons for this study could be estimated with a 95% CI that was ±8.3. The study size was viewed to have less sensitivity for a statistically successful difference in dichotomous outcome (e.g., smoking cessation as a secondary efficacy outcome), other than for possibly large differences. For example, if the control arm outcome was 12% (3/25 defined as successes), then the experimental arm would have to have 36% (8/50) or more (computation based on exact CI) to be statistically successful.

All analyses followed the intent-to-treat principle without imputation for missing outcome data, and participants with missing data at an assessment were considered to be smoking for that assessment.

The primary efficacy endpoint was based on the cigarette score with primary comparisons for this endpoint between each active treatment arm and the associated placebo arm.

Sensitivity analyses included the assessment of the contribution of interaction terms and effect modification analyses. The sensitivity analyses were performed for both the primary efficacy endpoint and the continuous abstinence from Week 5 to Week 8 endpoint.

The placebo and treatment arms were compared using an analysis of variance (ANOVA) model with fixed effects for treatment arm (2 levels) and BMI class as a potential confounder (3 levels), and a covariate of baseline cigarettes. The least-squares (LS) means, LS means differences, and their respective 95% CIs were reported. The absence of placebo arm differences was regarded as justification for pooling, with the purpose of increased statistical sensitivity in this small Phase 2b study and, in that case, the efficacy analyses were repeated using the pooled placebo arms.

For the initial quit rate and continuous abstinence from Week 5 to Week 8 endpoints, comparisons between the placebo arms were also performed, and if no placebo arm differences, the pooled placebo arm was used for comparisons to the 4 cytisinicline arms.

## Results

### Demographics and Smoking History

**Figure 1.**
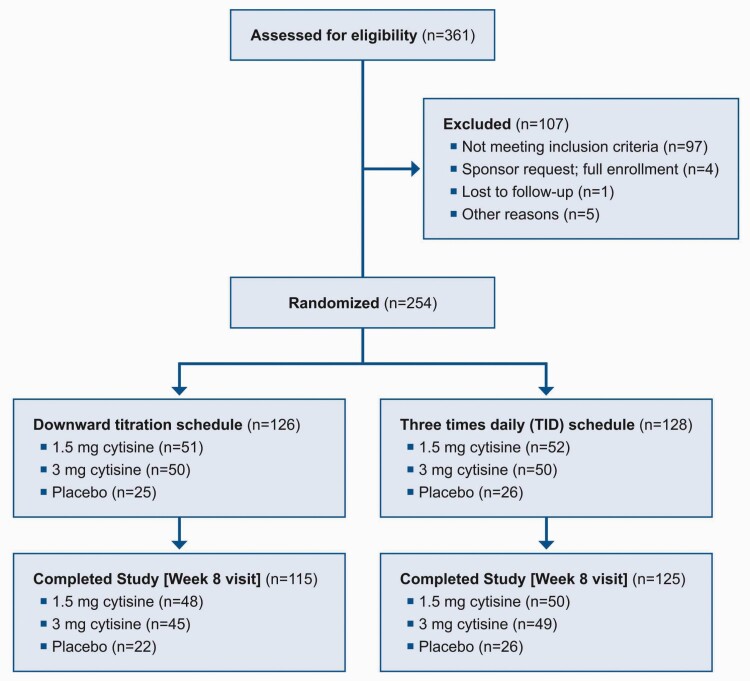
Participant disposition.

Participant disposition is shown in Figure 1. A total of 254 participants were randomized. The trial’s visit schedule ran from 20 November 2018 until completion on 23 April 2019.

Demographics and smoking history at baseline (Table 1) were generally similar across treatment arms.

**Table 1.** Demographics and smoking history at baseline

	Cytisinicline Downward Titration Schedule		Cytisinicline TID Schedule			
Parameter – *n* (%)	1.5 mg (*N* = 51)	3 mg (*N* = 50)	1.5 mg (*N* = 52)	3 mg (*N* = 50)	Pooled Placebo (*N* = 51)	All Participants (*N* = 254)
Sex						
Female	28 (54.9)	20 (40.0)	29 (55.8)	25 (50.0)	31 (60.8)	133 (52.4)
Race						
White	43 (84.3)	40 (80.0)	37 (71.2)	41 (82.0)	39 (76.5)	200 (78.7)
Black or African American	7 (13.7)	9 (18.0)	13 (25.0)	7 (14.0)	10 (19.6)	46 (18.1)
Asian	0	0	1 (1.9)	1 (2.0)	1 (2.0)	3 (1.2)
Other	1 (2.0)	1 (2.0)	1 (1.9)	1 (2.0)	1 (2.0)	5 (2.0)
Age (years)						
Mean (SD)	49.8 (11.46)	50.0 (13.20)	47.0 (13.67)	46.3 (12.88)	49.0 (13.94)	48.4 (13.04)
BMI (kg/m^2^)						
Mean (SD)	27.6 (4.31)	27.8 (4.10)	27.9 (4.17)	27.1 (4.12)	27.9 (4.79)	27.7 (4.28)
Duration of smoking (yrs)						
Mean (SD)	33.27 (12.054)	33.17 (15.158)	30.91 (13.734)	29.97 (13.374)	32.95 (14.134)	32.05 (13.682)
Average number of cigarettes smoked per day past 30 days						
Mean (SD)	18.5 (5.96)	17.7 (5.12)	19.0 (7.52)	17.4 (5.01)	18.2 (6.07)	18.2 (6.00)
Number of previous quit attempts						
Median	4.0	2.5	3.0	3.0	4.0	3.0
Min, max	1, 50	1, 20	1, 20	1, 12	1, 25	1, 50
Previous quit attempt treatments ever used (>10 of all participants)^1^						
Nicotine patch	23 (45.1)	19 (38.0)	27 (51.9)	25 (50.0)	28 (54.9)	122 (48.0)
Varenicline	21 (41.2)	13 (26.0)	21 (40.4)	18 (36.0)	19 (37.3)	92 (36.2)
Nicotine gum	20 (39.2)	12 (24.0)	20 (38.5)	16 (32.0)	24 (47.1)	92 (36.2)
Electronic cigarette	15 (29.4)	11 (22.0)	19 (36.5)	13 (26.0)	18 (35.3)	76 (29.9)
Bupropion	9 (17.6)	3 (6.0)	9 (17.3)	7 (14.0)	12 (23.5)	40 (15.7)
Other	6 (11.8)	4 (8.0)	8 (15.4)	5 (10.0)	5 (9.8)	28 (11.0)
Cessation advice from medical practitioner	3 (5.9)	6 (12.0)	7 (13.5)	4 (8.0)	6 (11.8)	26 (10.2)

TID = 3 times daily; BMI = body mass index; max = maximum; min = minimum; SD = standard deviation; yrs = years.

^1^Multiple occurrences of the same treatment within a participant were counted only once.

Twelve (9.5%) participants in the titration arms discontinued treatment early: 4 (7.8%) in the cytisinicline 1.5-mg arm, 6 (12.0%) in the cytisinicline 3-mg arm, and 2 (8.0%) in the placebo arm. Of these 12 participants who discontinued treatment early, 8 participants discontinued due to withdrawal of consent, 3 due to an AE, and 1 was lost to follow-up. On the TID schedule, 3 (2.3%) participants discontinued treatment early: 1 (1.9%) in the cytisinicline 1.5-mg arm, 1 (2.0%) in the cytisinicline 3-mg arm, and 1 (3.8%) in the placebo arm. Of the 3 participants who discontinued treatment early, 2 participants discontinued due to an AE, and 1 discontinued by missing the Day 20 visit, which led to not receiving study drug to Day 25. Participants who discontinued treatment early were encouraged to remain in the study and complete the Week 8 visit. On both schedules, approximately 94% of participants completed the study (i.e., completed the Week 8 visit): 115 (91.3%) participants on the titration schedule and 125 (97.7%) on the TID schedule.

### Compliance with Study Drug Administration and Study Diary Completion

All participants were treated according to their randomized treatment assignment. Mean compliance with study drug administration was calculated for individual participants by dividing the number of doses taken by the number of doses prescribed. On the titration schedule, overall mean compliance was 94.9% and similar across treatment arms. 10.3% participants had compliance <90%. On the TID schedule, overall mean compliance was 98.2% and similar across treatment arms. 3.1% participants had compliance <90%.

Mean compliance with smoking diary completion was calculated for individual participants by dividing the number of completed diary entries by the number of scheduled diary entries. Completing the 7-day screening smoking diary was an inclusion criterion for study entry; thus, participants had 100% compliance. For the titration schedule, mean compliance during the 25-day treatment period was 96.9% in the cytisinicline 1.5-mg arm, 94.6% in the cytisinicline 3.0-mg arm, and 95.7% in the placebo arm. All participants on the TID schedule had 100% compliance with the smoking diary during the 25-day treatment period.

### Key Endpoints

Results from the ANOVA sensitivity analysis for the percentage of expected cigarettes smoked in the placebo arms of each schedule demonstrated there was no statistically significant difference between the placebo arms. Similar sensitivity analyses showed no statistically significant differences between placebo arms of each schedule for initial Week 4 abstinence (quit) rates and for Week 5–8 continuous abstinence rates. Therefore, the placebo arms were pooled according to *a priori* specifications. ANOVA analyses of the proportion of expected cigarettes smoked (primary outcome) indicated that the differences between cytisinicline and placebo for both cytisinicline arms (1.5 mg and 3 mg) on the titration schedule and the cytisinicline 1.5-mg arm on the TID schedule were statistically significant and that the difference in this measure between cytisinicline and placebo with the cytisinicline 3-mg TID arm approached statistical significance (Table 2). Individual ANOVA analyses of the proportion of expected cigarettes smoked as separate analyses for the titration schedule and TID schedule, without pooling placebo arms, indicated statistically significant differences between cytisinicline and placebo only in comparison of the titration schedules (Supplemental Tables 1 and 2).

**Table 2.** Percentage of expected cigarettes smoked versus pooled placebo

	Cytisinicline Downward Titration Schedule		Cytisinicline TID Schedule		
Parameter	1.5 mg (*N* = 51)	3 mg (*N* = 50)	1.5 mg (*N* = 52)	3 mg (*N* = 50)	Pooled Placebo (*N* = 51)
Percentage of expected cigarettes smoked					
Mean (SD)	26.7 (20.1)	25.1 (18.1)	29.5 (24.4)	32.5 (21.4)	41.5 (26.5)
Median	19.7	22.0	21.0	26.4	37.7
Min, max	4.8, 83.6	1.4, 78.6	3.5, 96.4	0.6, 97.0	1.6, 126.9
LS mean (SE)	26.7 (3.1)	25.1 (3.1)	29.3 (3.3)	32.4 (3.4)	41.7 (3.4)
95% CI	(20.6, 32.7)	(19.0, 31.2)	(22.8, 35.8)	(25.8, 39.0)	(35.1, 48.3)
LS mean difference vs placebo	−14.6	−16.2	−12.4	−9.3	
95% CI	(−23.2, −6.0)	(−24.9, −7.5)	(−21.7, −3.1)	(−18.7, 0.1)	
*p*-value (vs. placebo)	0.0010	0.0003	0.0091	0.0518	

TID = 3 times daily; *N* = Number of safety participants in the specified treatment arm; LS = Least Squares; SD = Standard Deviation; SE = Standard Error; CI = Confidence Interval.

**Figure 2.**
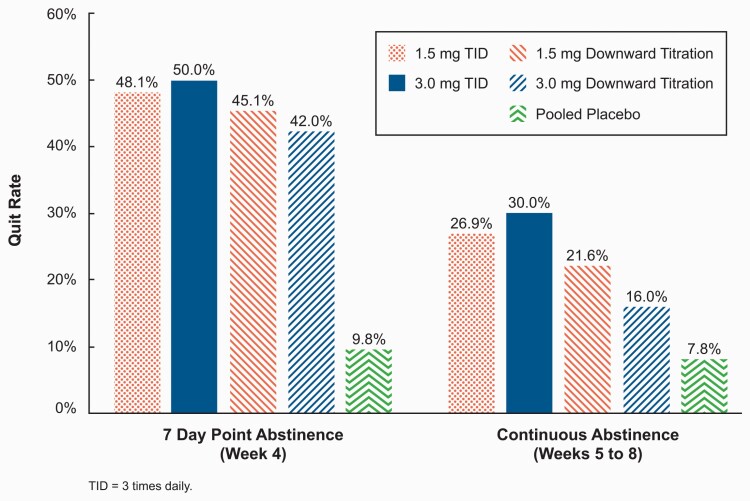
Abstinence rates verified by expired carbon monoxide.

As shown in Figure 2, CO-verified 7-day point-prevalence abstinence rates at EOT (Week 4; Day 21 to 27) were statistically significantly higher (*p* < 0.001) in all cytisinicline treatment arms compared with placebo: 45% and 42% for the 1.5-mg and 3-mg titration arms and 48% and 50% for the 1.5-mg and 3-mg TID arms, respectively, compared with 10% for the pooled placebo arms (12% and 8% for the titration and TID placebo arms, respectively). Based on CO-verified 7-day point prevalence abstinence rates at each Week 5, 6, 7, and 8 visits, the odds of quitting remained statistically significantly higher in each of the cytisinicline TID arms compared with placebo, even though the weekly quit rates reduced to 38% in the cytisinicline arms (Supplemental Table 3). The odds of quitting were not statistically significantly higher in the titration arms versus placebo with quit rates lower and reduced to below 30%.

Continuous abstinence for the 4 weeks from Week 5 through Week 8 assessment is also shown in Figure 2. The TID schedule for cytisinicline had statistically significantly higher continuous abstinence rates compared with placebo, with participants in the cytisinicline 3-mg TID arm having the highest continual abstinence rate at 30% compared with 8% for pooled placebo (8% for both of the titration and TID placebo arms). Participants in the 3-mg TID arm had an odds ratio (OR) of 5.04 (95% CI: 1.42, 22.32) for continuous abstinence from Week 5 to Week 8, compared with placebo. The OR for the 1.5-mg TID arm was 4.33 (95% CI: 1.21, 19.30). On the titration schedule, the ORs for the 1.5- and 3-mg arms were 3.23 (95% CI: 0.86, 14.85) and 2.24 (95% CI: 0.55, 10.82), respectively.

No participant experienced a serious AE (SAE). No new or unexpected AEs were identified during the study. Most AEs were mild or moderate in intensity; only 2 (0.8%) were severe in intensity, and both were unrelated to the study drug. The most common AEs in the study were upper respiratory tract infection (7.9%), abnormal dreams (7.5%), and nausea (6.7%). About half of the AEs (52.9%) were determined by the investigators to be “not related” or “unlikely to be related” to the study drug. Most (95.3%) AEs did not result in a change in dosing, and the participant was considered recovered (88.2%) during the study. A total of 3 participants who received cytisinicline and 2 participants who received placebo discontinued study treatment early due to an AEs. Adverse events experienced by ≥2% of participants in the trial are summarized in Table 3.

**Table 3.** Treatment-emergent adverse events experienced by ≥2% of participants in the trial

	Cytisinicline Downward Titration Schedule		Cytisinicline TID Schedule			
Parameter – *n* (%)	1.5 mg (*N* = 51)	3 mg (*N* = 50)	1.5 mg (*N* = 52)	3 mg (*N* = 50)	Pooled Placebo (*N* = 51)	All Participants (*N* = 254)
Upper respiratory tract infection	3 (5.9)	2 (4.0)	5 (9.6)	3 (6.0)	7 (13.7)	20 (7.9)
Abnormal dreams	4 (7.8)	7 (14.0)	4 (7.7)	3 (6.0)	1 (2.0)	19 (7.5)
Nausea	5 (9.8)	3 (6.0)	1 (1.9)	3 (6.0)	5 (9.8)	17 (6.7)
Insomnia	3 (5.9)	4 (8.0)	4 (7.7)	3 (6.0)	1 (2.0)	15 (5.9)
Headache	1 (2.0)	1 (2.0)	6 (11.5)	2 (4.0)	2 (3.9)	12 (4.7)
Fatigue	1 (2.0)	2 (4.0)	3 (5.8)	1 (2.0)	2 (3.9)	9 (3.5)
Nasopharyngitis	2 (3.9)	2 (4.0)	0	2 (4.0)	1 (2.0)	7 (2.8)
Gastroenteritis	1 (2.0)	0	2 (3.8)	1 (2.0)	2 (3.9)	6 (2.4)
Anxiety	1 (2.0)	1 (2.0)	0	1 (2.0)	3 (5.9)	6 (2.4)
Vomiting	2 (3.9)	2 (4.0)	0	1 (2.0)	0	5 (2.0)
Constipation	0	0	1 (1.9)	3 (6.0)	1 (2.0)	5 (2.0)
Diarrhoea	2 (3.9)	1 (2.0)	0	0	2 (3.9)	5 (2.0)
Hypertension	2 (3.9)	0	2 (3.8)	0	1 (2.0)	5 (2.0)

TID = 3 times daily; *N*: Number of safety participants in the specified treatment arm; *n*: Number of participants with data available in the specified treatment arm.

Treatment-emergent AE was defined as any AE that was new in onset or was aggravated in severity or frequency after the first dose of study drug up to and including the last visit of the study.

Adverse events were coded using Medical Dictionary for Regulatory Activities (MedDRA) version 21.0.

If a participant experienced more than 1 finding within a given system organ class, that participant was counted only once for that system organ class. If a participant experienced more than 1 finding with a given preferred term, that participant was counted only once for that preferred term.

## Discussion

Overall, cytisinicline treatment resulted in larger reductions in the expected number of cigarettes smoked during treatment versus placebo (the primary outcome measure), higher abstinence rates by the end of treatment, and higher continuous abstinence rates at 4 weeks post-treatment versus placebo. The TID schedule for cytisinicline had statistically significantly higher continuous abstinence rates at 27% and 30% for 1.5-mg or 3-mg cytisinicline doses, respectively, compared with 8% for placebo. There were no safety concerns with either 1.5-mg or 3-mg cytisinicline, regardless of administration schedule. The incidence of nausea, vomiting, abnormal dreams, insomnia, and headache was low, and the incidence of some AEs, such as nausea, was similar to placebo rates. The demonstration of tolerability of cytisinicline 3 mg, the high initial quit rates after TID treatment (50%), and continued abstinence post-treatment (30%) without further treatment supports further development of the higher 3-mg dose, with a simplified TID administration as a marketed product for smoking cessation. Longer than 25-day treatment periods with cytisinicline may also be of benefit to reduce post-treatment smoking relapses.

This study population smoked cigarettes for a mean number of 32 years with an average of 18 cigarettes per day and had attempted to quit smoking in the past with an average of 4.5 previous quit attempts; thus, representing a population of smokers who were highly dependent on smoking.

The international EAGLES clinical trial,^12^ comparing varenicline, bupropion, and nicotine patch to placebo, in general, reported lower quitting success rates for participants in the U.S versus participants outside the U.S., regardless of treatment.^15^ The lower success rates in the U.S. highlights the need for additional, and potentially more effective, treatments to help these dependent smokers quit.

Results from the primary analyses demonstrated significant reductions in the percentage of expected cigarettes smoked during the 25-day cytisinicline treatment versus placebo. Although the reported reduction in cigarettes smoked appeared lower in the TID schedule arms, continuous abstinence rates using biomarker verification were higher in the TID schedule arms. However, by Week 8, abstinence failures were observed by both reporting of cigarettes smoked and by higher expired CO levels, which may indicate that longer cytisinicline treatment is needed to maintain smoking abstinence.

Cytisinicline treatment at all dosing and administration schedules was well tolerated with few AEs and minimal treatment discontinuations. There were no safety concerns following cytisinicline dosing in the 1.5- or 3–mg arms on either schedule. This finding is especially notable for nausea. A recent analysis of nausea rates in 2 smoking cessation clinical trials conducted at multiple sites in the U.S. and Canada found that early nausea was indirectly associated with lower cessation rates at multiple timepoints as a result of reduced varenicline adherence (ORs ranging from 0.92–0.94; 95% CI between 0.83–0.99).^16^ A prior analysis of 6 varenicline clinical trials found that nausea was the most common AE, occurring in 28.1% of participants.^17^ In contrast, in the ORCA-1 study described here, fewer than 10% of participants reported nausea as an AE (Table 3). This lower rate of nausea might improve adherence to cytisinicline, and if verified, may result in improved quit rates.

Based on regulatory discussions and overall ORCA-1 results for pursuing a potential market approval for cytisinicline in the U.S., the 3-mg TID dose/schedule was selected for further evaluation in a Phase 3 program. This was supported by 1) the slightly higher compliance for the more simplified TID scheduling, 2) the statistically significant higher odds of success for continued abstinence with the 3-mg TID schedule and 3) the continued excellent safety profile at the higher 3-mg dosing. Longer treatment schedules are also being evaluated to reduce potential relapses in smoking.

### Strengths

The primary strength of this study is its blinded, placebo-controlled, randomized study design that reduces bias. The treatments were all randomly assigned and blinded within each schedule. As the titration and simplified TID schedules had different tablet administrations, the corresponding placebo layout was incorporated so that placebo bias, compliance, and AEs could be accurately evaluated. Continued smoking abstinence from Week 5 through Week 8 with weekly biochemical verification is an objective endpoint and an acceptable primary endpoint measure for regulatory approval by FDA. Behavioral support has also been shown to be effective in helping smokers to quit, and all study participants received behavioral support to aid their smoking cessation attempt.

### Limitations

The primary endpoint for this study was based on self-reporting of daily cigarettes smoked, which was a subjective measure for the reduction in cigarettes smoked.

## Supplementary Material

A Contributorship Form detailing each author’s specific involvement with this content, as well as any supplementary data, are available online at https://academic.oup.com/ntr.

ntab073_suppl_Supplementary_Tables

ntab073_suppl_Supplementary_Taxonomy_Form
