# Supplementary figures and images for: A Multicenter, Double-Blind, Randomized, Placebo-Controlled Phase 2b Trial of Cytisinicline in Adult Smokers (The ORCA-1 Trial)
Source: Nicotine Tob Res. 2021 Apr 12;23(10):1656–63. doi: 10.1093/ntr/ntab073 (PMC8403245; doi:10.1093/ntr/ntab073)

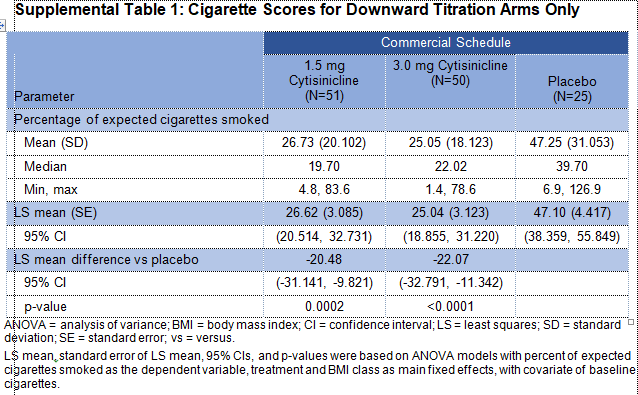


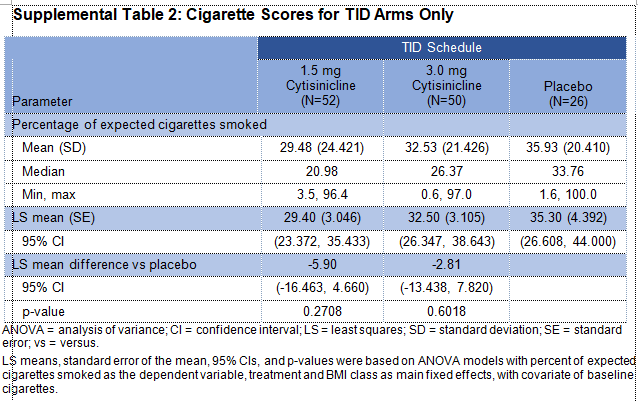


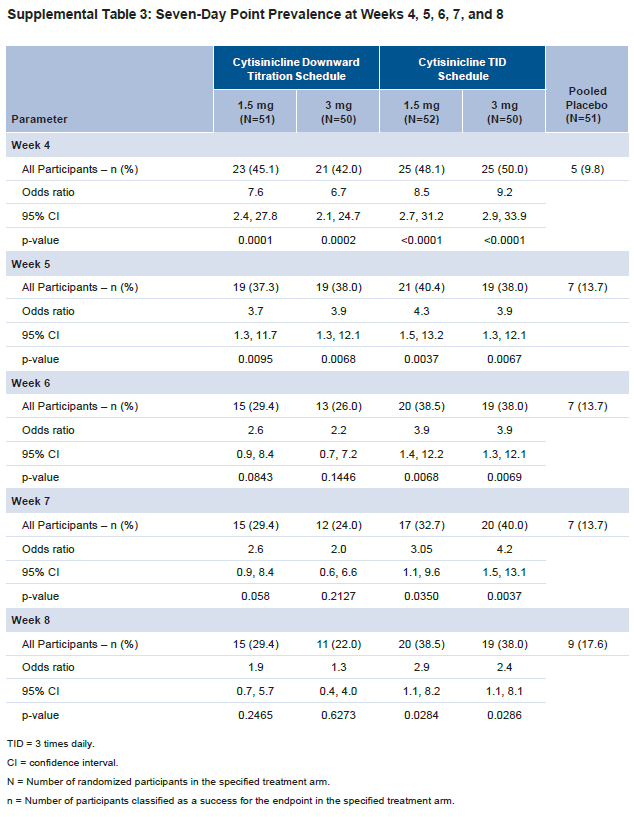

Supplement: ntab073_suppl_Supplementary_Tables [file ntab073_suppl_supplementary_tables.docx]
